# Signatures of selection for bonamiosis resistance in European flat oyster (*Ostrea edulis*): New genomic tools for breeding programs and management of natural resources

**DOI:** 10.1111/eva.12832

**Published:** 2019-07-05

**Authors:** Manuel Vera, Belén G. Pardo, Asunción Cao, Román Vilas, Carlos Fernández, Andrés Blanco, Alejandro P. Gutierrez, Tim P. Bean, Ross D. Houston, Antonio Villalba, Paulino Martínez

**Affiliations:** ^1^ Department of Zoology, Genetics and Physical Anthropology, ACUIGEN group, Faculty of Veterinary Universidade de Santiago de Compostela Lugo Spain; ^2^ Instituto de Acuicultura Universidade de Santiago de Compostela Lugo Spain; ^3^ Centro de Investigacións Mariñas (CIMA) Consellería do Mar, Xunta de Galicia Pontevedra Spain; ^4^ The Roslin Institute and Royal (Dick) School of Veterinary Studies University of Edinburgh Midlothian UK; ^5^ Departamento de Ciencias de la Vida Universidad de Alcalá Madrid Spain; ^6^ Research Centre for Experimental Marine Biology and Biotechnology (PIE) University of the Basque Country (UPV/EHU) Basque Country Spain

**Keywords:** *Bonamia ostreae*, candidate genes, disease resistance, divergent selection, genetic traceability, *Ostrea edulis*, SNP array

## Abstract

The European flat oyster (*Ostrea edulis*) is a highly appreciated mollusk with an important aquaculture production throughout the 20th century, in addition to playing an important role on coastal ecosystems. Overexploitation of natural beds, habitat degradation, introduction of non‐native species, and epidemic outbreaks have severely affected this important resource, particularly, the protozoan parasite *Bonamia ostreae,* which is the main concern affecting its production and conservation. In order to identify genomic regions and markers potentially associated with bonamiosis resistance, six oyster beds distributed throughout the European Atlantic coast were sampled. Three of them have been exposed to this parasite since the early 1980s and showed some degree of innate resistance (long‐term affected group, LTA), while the other three were free of *B*. *ostreae* at least until sampling date (naïve group, NV). A total of 14,065 SNPs were analyzed, including 37 markers from candidate genes and 14,028 from a medium‐density SNP array. Gene diversity was similar between LTA and NV groups suggesting no genetic erosion due to long‐term exposure to the parasite, and three population clusters were detected using the whole dataset. Tests for divergent selection between NV and LTA groups detected the presence of a very consistent set of 22 markers, located within a putative single genomic region, which suggests the presence of a major quantitative trait locus associated with *B. ostreae* resistance. Moreover, 324 outlier loci associated with factors other than bonamiosis were identified allowing fully discrimination of all the oyster beds. A practical tool which included the 84 highest discriminative markers for tracing *O. edulis* populations was developed and tested with empirical data. Results reported herein could assist the production of stocks with improved resistance to bonamiosis and facilitate the management of oyster beds for recovery production and ecosystem services provided by this species.

## 1 INTRODUCTION

The flat oyster *Ostrea edulis* has been one of the most appreciated seafood products since the Roman times and represented an important aquaculture species in Europe throughout the 20th century. Additionally, oyster reefs play an important ecosystem role providing multiple benefits such as water filtration, food and habitat for other animals, shoreline stabilization, carbon burial, nutrient regeneration, and coastal fisheries (Grabowski & Peterson, 2007). *Ostrea edulis* habitats are endangered in Europe and have been identified as a priority for protection and restoration in the European MPAs (OSPAR Commission, 2011).

Overexploitation of natural beds, introduction of non‐native oysters, adverse effects of climate change and specific epidemic outbreaks, such as the “shell disease” caused by the fungus *Ostracoblabe implexa* (Alderman & Jones, 1971), the protist *Marteilia refringens* (Grizel et al., 1974) and the protozoan parasite *Bonamia ostreae* (Pichot, Comps, Tigé, Grizel, & Rabouin, 1980), have determined the decline of flat oyster wild production down to marginal figures in many European areas (a peak of 32,995 t in 1961 to 3,120 t in 2016; http://www.fao.org/fishery/statistics/global-production/es). Bonamiosis, endemic in many areas of flat oyster distribution, is currently the main concern for flat oyster production and the main contributory factor to its decline (Engelsma, Culloty, Lynch, Arzul, & Carnegie, 2014); therefore, effective control of this parasitism is considered essential for the recovery of the species. Successful breeding programs aiming to obtain resistant strains have been accomplished in several oyster species including different pathogens, such as the ostreid herpesvirus 1, *Haplosporidium nelsoni*, *Perkinsus olseni*, *Roseobacter crassostreae*, *Marteilia sydneyi* or *B. ostreae*, by selecting survivors after long exposure in heavily affected areas (Beattie, Davis, Downing, & Chew, 1988; Dégremont, Nourry, & Maurouard, 2015; Dove, Nell, & O’Connor, 2013; Ford & Haskin, 1987; Frank‐Lawale, Allen, & Degremont, 2014; Lynch, Flannery, Hugh‐Jones, Hugh‐Jones, & Culloty, 2014; Ragone Calvo, Calvo, & Burreson, 2003). The procedure usually involves the evaluation of populations in the field to identify resistant individuals to be used as breeders for the next generation and can take up to 4–5 years. This has limited its efficacy due to the inability to maintain constant selection pressure when disease exposure is low or absent. Accordingly, detection of quantitative trait loci (QTL) and markers associated with resistance to bonamiosis could greatly accelerate the selection of breeders making the process much more efficient. Such resistance markers have been successfully found for some diseases in mollusks (He, Yu, Bao, Zhang, & Guo, 2012; Meistertzheim et al., 2014; Nie, Yue, & Liu, 2015; Nikapitiya et al., 2014; Normand et al., 2014; Raftos, Kuchel, Aladaile, & Butt, 2014; Schmitt, Santini, Vergnes, Degremont, & de Lorgeril, 2013), and particularly, several QTL for resistance to bonamiosis have been identified in flat oyster (Harrang et al., 2015; Lallias et al., 2009). These studies typically used low–medium density genetic maps and a small number of families and were able to identify some QTLs explaining up to 17% of the phenotypic variance in the trait (Harrang et al., 2015; Lallias et al., 2009). However, this information, although useful, should be considered preliminary since it is far from elucidating the genetic architecture of bonamiosis resistance in the oyster populations of the Atlantic area, considering the low number of analyzed families. Moreover, the detected QTL should be considered as suggestive, since they were identified at chromosome level (*p* < 0.05) and in only one or two families.

Recently, a gene expression study using a flat oyster hemocyte oligo‐microarray (Pardo et al., 2016) enabled the identification of specific genes and enriched pathways differentially expressed (DE) between two flat oyster stocks with differential susceptibility to bonamiosis: a naïve one (NV) from a free‐bonamiosis area and a long‐term affected one (LTA), which had shown some resistance to *B. ostreae* (da Silva, Fuentes, & Villalba, 2005; Villalba, da Silva, & Fuentes, 2007). The LTA stock showed a substantially more intense and rapid response to infection than the NV, with the activation of several signal transduction pathways early after challenge with *B. ostreae*, likely representing an important factor for the resistance observed (Ronza et al., 2018). Furthermore, cell–extracellular matrix (ECM) interactions and proteases inhibitors were essential in the defense response of LTA oysters, while the broad down‐regulation of histone‐related genes in NV oysters suggested a fundamental role of these proteins during the infection process. The set of DE genes detected in this study and the proper oyster database, where a substantial number of gene‐linked markers are available (Pardo et al., 2016), represent a useful repository to build upon the identification of genetic markers associated with candidate genes linked to bonamiosis resistance.

Marine species typically show low structuring due to high effective population sizes and high gene flow facilitated by the absence of physical barriers, which lead to high genomic homogenization across populations (Vandamme et al., 2014; Vilas et al., 2015). However, habitat shifts (ecotones) and oceanic currents/ fronts have been demonstrated to act as practical barriers (Blanco‐González, Knutsen, & Jorde, 2016; Vera et al., 2016), and particularly, natural selection in response to abiotic and biotic factors has been demonstrated to actively shaping specific genomic regions related to adaption even in a context of high gene flow (Vandamme et al., 2014; Vilas, Bouza, Vera, Millán, & Martínez, 2010; Vilas et al., 2015). The ability to distinguish between neutral and adaptive genetic variation has become a central issue in evolutionary biology, as it would allow the understanding of population structure in both historical/demographic and adaptive terms (Bernatchez, 2016; Nielsen, Hemmer‐Hansen, Larsen, & Bekkevold, 2009), thus being an essential information for sustainable fisheries management. In turn, genetic diversity in the wild represents the raw material for the foundation of aquaculture broodstocks and consequently a reference to identify selection signatures for targeted traits in farmed populations which can be detected via genome scanning (e.g., Liu et al., 2017). Several European flat oyster populations have been subjected to the selective pressure of bonamiosis for many generations, while other oyster populations have never been in contact with the parasite. This scenario raises the hypothesis that specific genomic regions related to parasite resistance have been under selection and therefore modified by this selective pressure. In fact, increase of resistance to *B. ostreae* due to natural selection associated with long exposure of oyster populations to this parasite was supported by field experiments (Elston, Kent, & Wilkinson, 1987; da Silva et al., 2005). Consequently, a genetic comparison of several LTA vs. NV oyster populations originating from different sources and geographical regions, thus being essentially considered as independent replicates, could aid to identify those genomic regions subjected to selection.

In the current study, we characterized and compared two sets of three LTA vs. three NV flat oyster populations collected throughout the European Atlantic area and involving the three genetically differentiated regions previously reported (Vera et al., 2016) to identify candidate genomic regions related to bonamiosis resistance by using two different strategies: (a) a panel of validated genetic markers from DE candidate genes between LTA and NV populations; (b) a large panel of SNPs covering the whole genome at a high density (1 SNP per 80 kb on average). A consistent set of 28 closely linked genetic markers from a putative single genomic region was identified which is suggestive of a strong QTL differentiating both population sets.

## 2 MATERIAL AND METHODS

### 2.1 Sampling

**Figure 1:**
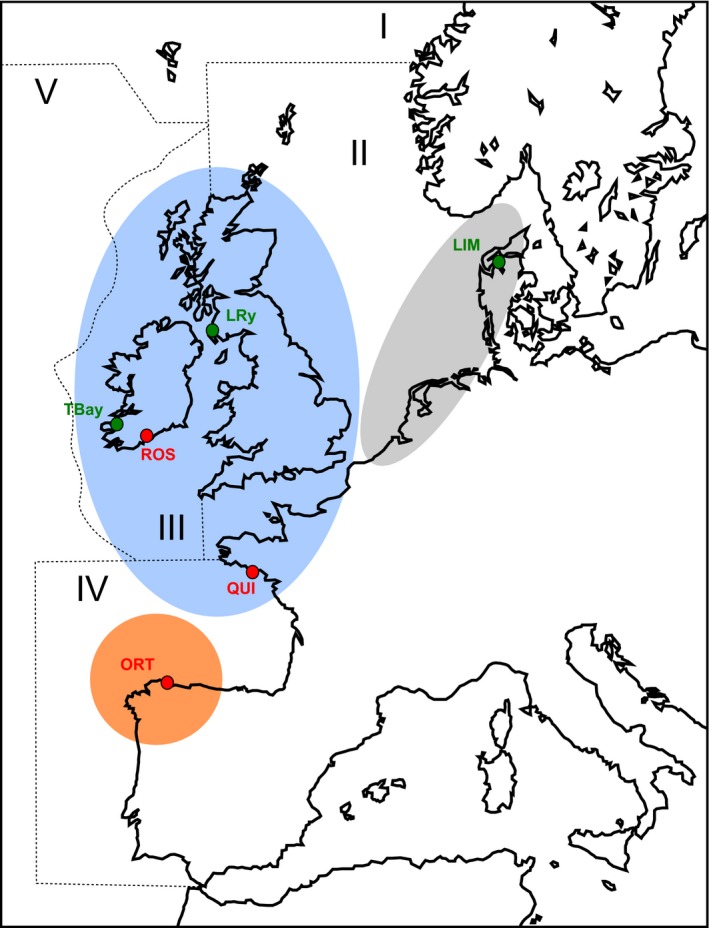
Geographical situation of the *Ostrea edulis* locations in the present study. Dotted lines and roman numbers show the OSPAR regions. The three population genetic clusters previously described by Vera et al. (2016) are also represented (orange circle: Spanish cluster; blue ellipse: Irish/British/French cluster; gray ellipse: Dutch/Danish cluster). Locations long‐term exposed to Bonamia are indicated in red, while naïve locations are indicated in green. Location codes are shown in Table 1

A total of 199 individuals from six oysters beds distributed throughout the European Atlantic coast coming from the three genetically differentiated regions previously reported for the species (i.e., Spanish cluster, Irish/British/French cluster, and Dutch/Danish cluster; Vera et al., 2016) and included in three different OSPAR regions (Region II: Greater North Sea; III: Celtic Seas; IV: Bay of Biscay and Iberian Coast) were sampled in 2010 and stored in 100% ethanol for DNA extraction and analyses (Table 1, Figure 1). These three flat oyster genetic regions seem to show high connectivity due to larval advection according to their low differentiation (global *F*_ST_ = 0.0079), and this low but significant differentiation has been suggested to be driven by oceanic fronts present in the region (Vera et al., 2016). Three of the oyster beds, Ortigueira (ORT), Quiberon (QUI), and Rossmore (ROS), had been exposed to *B*. *ostreae* since the early 1980s (McArdle, McKiernan, Foley, & Jones, 1991; Pichot et al., 1980; Polanco, Montes, Outon, & Melendez, 1984), which involves ca. 10–15 generations up to 2010, when oysters for this study were collected, assuming 2–3 years per generation; oysters from these long‐term affected (denoted LTA) stocks have shown some resistance to the parasite (Lynch et al., 2014; da Silva et al., 2005). Specifically, the ROS population has been under a breeding program for bonamiosis resistance, managed by Atlantic Shellfish Ltd. close to Cork Harbour, Ireland (Lynch et al., 2014), which does not imply an intensive selective pressure because of both its dependence on the variable natural parasite pressure and the recruitment of oyster breeders from natural beds. The other three beds, Limfjord (LIM), Loch Ryan (LRy), and Tralee Bay (TBay), had kept free of *B*. *ostreae* until sampling date (naïve—denoted NV), according to periodical surveys since the 1980s (Flannery, 2014; Laing, Dunn, Peeler, Feist, & Longshaw, 2014; Madsen, Kamp, & Mellergaard, 2013), as required to keep their official status of *B. ostreae*‐free areas, which was operative when oysters were collected for this study. The distribution of *B*. *ostreae* across the European Atlantic coast is quite patchy, and although larvae have demonstrated to be capable of a mild infection by the parasite (Arzul et al., 2011), this does not ensure the settling of the parasite in other areas (Flannery, Lynch, & Culloty, 2016). Nevertheless, oyster movements by oystermen from affected‐ into previously non‐affected areas have likely been the most effective way for bonamiosis spreading through the Atlantic coast. This would explain the presence or absence of the parasite in relatively close populations such as TBay and ROS**.** Long‐term surveys performed throughout the Irish coast to assess the *Bonamia*‐free status or the occurrence of *B. ostreae* have shown through the years a trickle of new detections of the parasite in Irish Bays, thus losing their consideration of *Bonamia*‐free Bays (Culloty & Mulcahy, 2007). In addition, in accordance with their different parasite pressure, oysters coming from populations with LTA and NV profile (ORT vs. LIM) showed very different hemocyte gene expression profiles after challenging with the parasite compatible with their more tolerant and naive conditions, respectively (Ronza et al., 2018).

**Table 1:** Flat oyster beds analyzed in the present study

Location	Code	Country	Geographical Coordinates	*N*	N_CAND_	N_ARRAY_	Bonamiosis status	Na	Ho	He	*F*_IS_
Rossmore	ROS	Ireland	51º 50' 13'' *N* 08º 16' 38'' W	33	29	16	Long‐term affected	1.84	0.314	0.309	−0.016
Ortigueira	ORT	Spain	43º 42' 36'' *N* 07º 52' 18'' W	35	30	16	Long‐term affected	1.87	0.315	0.309	−0.019
Quiberon	QUI	France	47º 28' 40'' *N* 03º 07' 13'' W	30	29	15	Long‐term affected	1.85	0.314	0.313	−0.003
Limfjord	LIM	Denmark	56º 55' 28'' *N* 08º 58' 51'' E	41	29	16	Naïve	1.70	0.386	0.334	−0.156
Loch Ryan	LRy	Scotland	54º 57' 12'' *N* 05º 02' 12'' W	30	30	16	Naïve	1.86	0.305	0.304	−0.003
Tralee Bay	TBay	Ireland	52º 17' 28'' *N* 09º 56' 14'' W	30	29	16	Naïve	1.86	0.316	0.311	−0.016

Note

*F*_IS_, intrapopulation fixation index; He, expected heterozygosity; Ho, observed heterozygosity; *N*, total number of individuals analyzed; Na, mean number of alleles per locus; *N*_ARRAY_, number of individuals analyzed for ARRAY markers; *N*_CAND_, number of individuals analyzed for CAND markers.

### 2.2 DNA extraction and SNP validation and genotyping

Total DNA was extracted from gill tissue using the e.Z.N.A. E‐96 mollusk DNA kit (OMEGA Bio‐tech), following the manufacturer recommendations. Two different strategies were followed for single nucleotide polymorphisms (SNPs) selection and genotyping to identify genetic markers associated with resistance to bonamiosis. The first one was focused on the search for SNPs associated with candidate (denoted “CAND” markers) differentially expressed genes (DEGs) between LTA vs. NV oysters (Ronza et al., 2018). The flat oyster transcriptome database available for the species (Pardo et al., 2016) and the list of the 837 DEG previously reported by Ronza et al. (2018) were the starting point for SNPs selection. This selection was performed according to technical features (SNP flaking regions—100bp—without additional SNPs) and the functional relevance of the genes related to bonamiosis response (Ronza et al., 2018). Selected SNPs were validated on a MassARRAY platform (Sequenom) at the USC node of the Spanish National Centre of Genotyping (CeGen ISCIII). The distinct mass of the extended primer identifies the SNP allele. MALDI‐TOF mass spectrometry analysis in an Autoflex spectrometer was used for allele scoring. All the 199 oysters sampled were genotyped for CAND SNPs (Table 1).

The second strategy for SNP genotyping was based on a high‐throughput screening of the flat oyster genome using a combined‐species medium‐density Affymetrix Axiom array designed and validated for Pacific oyster *(Crassostrea gigas*; 40,625 SNPs) and *O. edulis* (14,950 SNPs) (Gutierrez et al., 2017) (denoted “ARRAY” markers). Flat oyster DNA samples were delivered to the Edinburgh Genomics facility (The University of Edinburgh, UK) for genotyping. Only ARRAY markers genotyped in ≥80% of individuals were used for further analyses. A total of 95 individuals evenly distributed among the six oyster beds (~16 individuals/ bed) were used for ARRAY SNP genotyping (Table 1).

### 2.3 Genetic diversity and population structure

The mean number of alleles per locus (Na), observed (Ho), and expected (He) heterozygosity, departure from Hardy–Weinberg equilibrium (HWE) and the estimators of Wright (1951) F‐statistics at each locus were calculated using GENEPOP v4.0 (Rousset, 2008) and ARLEQUIN v3.5 (Excoffier & Lischer, 2010). Linkage disequilibrium was checked with exact tests to look for genotyping association using GENEPOP v4.0, and they were specifically applied to check linkage among loci related to bonamiosis resistance.

Pairwise *F*_ST_ values between oyster beds were calculated with ARLEQUIN v3.5 using 10,000 permutations to test for significance. Genetic subdivision was explored using an approach that does not require defining populations a priori. In particular, a model‐based clustering approach, implemented in STRUCTURE v2.3.4 (Pritchard, Stephens, & Donnely, 2000), which constructs a number of genetic clusters (*K*) from a collection of individual multilocus genotypes and estimates for each individual the fraction of its genome that belongs to each cluster, was applied. A burn‐in period of 10,000 steps and 100,000 Monte Carlo replicates for different values of *K* ranging from 1 to 7 was used. For each *K* tested, five replicates were run to assess their reliability. After checking that *K* = 1 was not the most probable scenario (see Results), the STRUCTURE results were reanalyzed with the program STRUCTURE HARVESTER v0.3 (Earl & vonHoldt, 2012) to estimate the best *K* value between 2 and 7 following the ΔK method described by Evanno, Regnaut, and Goudet (2005). The software CLUMPP v1.1.2 (Jakobsson & Rosenberg, 2007) was used to estimate the most likely cluster membership coefficient for each individual using the different runs tested with the Full Search option. Discriminant analyses of principal components (DAPC) were run in ADEGENET package (Jombart & Ahmed, 2011; Jombart, Devillard, & Balloux, 2010) for the R platform (R Development Core Team, 2014; http://www.r-project.org). Data were transformed using PCA (principal component analysis), and a number of principal components (PC) and discriminant functions (DF) were retained to explain >90% of the variance.

### 2.4 Divergent selection for bonamiosis resistance: outlier detection and gene mining

Different statistical strategies were used to identify outlier loci subjected to selection. BAYESCAN v2.01, a method based on a Bayesian approach to identify outliers using F_ST_ values under an inland model (Foll & Gaggiotti, 2008), was run using 20 pilot runs, prior odds value of 10, with 100,000 iterations, burn‐in of 50,000 iterations and a sample size of 5,000. Two different scenarios were tested with BAYESCAN: (a) BY_IND_, where the six oyster beds were considered as individual populations; (b) BY_POOL_, where oyster beds were pooled according to their bonamiosis status (LTA vs. NV). The other approach, ARLEQUIN v3.5, identifies outliers using the FDIST F_ST_ method using a maximum likelihood approach (Beaumont & Nichols, 1996), but has the advantage of incorporating a priori information regarding population structure. This method was applied to investigate outlier loci under the two scenarios also tested with BAYESCAN: (a) ARL_IND_ and (b) ARL_POOL_, but additionally including a third one, ARL_HIER_, where a hierarchical island model was applied grouping oyster beds by their resistance to bonamiosis. All ARLEQUIN runs were performed with 25,000 simulations, 10 groups, and 100 demes per group. Strategies to identify outliers under selection can produce type I (false positive) or type II (false negative) errors. BAYESCAN follows a more conservative approach than ARLEQUIN (Narum & Hess, 2011), and accordingly, loci with a false discovery rate (FDR, *q*‐value) <0.05 with BAYESCAN were considered consistent outliers. ARLEQUIN has the tendency to produce a higher proportion of false positives, and therefore, loci at *p* < 0.01 were considered consistent outliers, while those at *p* < 0.05 suggestive outliers. Markers shared (or excluded) by the different methods and scenarios were assumed to be the most reliable ones (see Results).

All sequences including divergent outlier SNPs were compared against the *O. edulis* database (Pardo et al., 2016) and the *C. gigas* genome [v9 assembly, Genbank accession # GCA_000297895.1, Zhang et al., 2012] using BLASTn and BLASTx tools with default parameters and *e*‐value < 1e‐5 within NCBI database. Functional enrichment of the gene lists against the *C. gigas* transcriptome was undertaken with BLAST2GO (Conesa et al., 2005).

### 2.5 Testing population discriminative and individual allocation capability of SNPs

Outlier SNPs putatively related to other factors than bonamiosis resistance were identified by discarding, from the total outliers detected with all strategies, those detected in the LTA vs. NV comparisons, putatively associated with bonamiosis since this was the factor used for grouping the set of populations analyzed covering the whole Atlantic area. This subset of outlier SNPs was used to estimate their capability for discriminating populations and for allocating individuals to their populations. These outliers were ordered according to global *F*_ST_ from higher (more discriminant) to lower (less discriminant) values, and then, subsets of markers were set up by progressively adding one by one SNPs following the *F*_ST_ rank. The USEPOPINFO option of the program STRUCTURE 2.3.4 was used to define the six reference populations studied. Then, for each SNP subset and individual tested, the admixture coefficient *q* was obtained for each of the six reference populations. With this information, we estimated the proportion of individuals assigned to their population according to the highest *q* (among the six calculated) and the highest one was considered the correct allocation. Using this information, we also estimated the average *q* value for each population and subset of SNPs tested.

Finally, an assignment test using the Bayesian method of Rannala and Mountain (1997) implemented in GENECLASS 2.0 (Piry et al., 2004) was performed to check for the power of the SNPs to assign individuals to their populations.

## 3 RESULTS

### 3.1 SNP genotyping and genetic diversity

Among the 837 DEGs identified by Ronza et al. (2018), 411 contained reliable SNPs in the flat oyster database and 390 of them were consistently annotated. After filtering using criteria described above, 140 markers were preselected according to technical features and 77 finally selected attending to their putative functional relevance regarding bonamiosis response. Primer sequences, SNP position, allelic variants, and annotation for the 77 tested SNPs are shown in Table S1. Among them, a total of 37 were consistently genotyped in 199 oysters from the six oyster beds sampled with the Mass Array technology at the USC platform and validated according to their in silico information. For the ARRAY markers, 14,028 SNPs out of the 14,950 passed the quality filters (93.8%) and were genotyped in 95 individuals from the six oyster beds with an average genotyping success per individual of 97.2% (range 88.9–98.4). Summing up, a total number of 14,065 markers were analyzed in the present study.

A total of 971 monomorphic loci were detected among the 14,065 analyzed; some of them might be monomorphic due to the limited sampling in our study, but also they might be false positive SNPs from the SNP discovery pipeline based on RAD Sequencing of pooled DNA samples (Gutierrez et al., 2017). Additionally, other 37 SNPs showed missing genotypes in more than 80% of samples and were discarded. Accordingly, a set of 13,057 polymorphic SNPs, indeed the most relevant information for the comparison between LTA and NV populations, were used for genetic diversity analysis. Mean number of alleles per locus (Na) ranged from 1.70 in LIM to 1.87 in ORT (mean = 1.83 ± 0.03). Observed heterozygosity (Ho) ranged from 0.305 in LRy to 0.386 in LIM (mean = 0.325 ± 0.012), while expected heterozygosity (He) from 0.304 in LRy to 0.313 in QUI (mean = 0.313 ± 0.004) (Table 1). No significant differences in genetic diversity were observed between LTA and NV oyster beds for all estimates (*p* > 0.175 for all Mann–Whitney *U* tests performed for each estimator), suggesting no loss of genetic diversity in the whole genome due to the selection pressure caused by the parasite. *F*_IS_ values were not significant in all cases excluding LIM (*p* < 0.05) which showed a slight heterozygote excess (*F*_IS_ = −0.156, Table 1). The percentage of loci not conforming to HW equilibrium (*p* < 0.05) was lower than 5% in all locations, excluding LIM (8.2%; 1,080 loci).

### 3.2 Signals of selection for CAND markers

We looked for outliers under selection by comparing the six oyster populations according to different statistical procedures (BAYESCAN and ARLEQUIN) and grouping scenarios (individual‐IND, pooling‐POOL, hierarchical‐HIER; see Materials and Methods). CAND outliers were exclusively detected with ARLEQUIN (16 in any of the three scenarios tested: IND, POOL, HIER), and most of them were suggestive for balancing selection, excluding Oed_rep_c4836_2869e and Oed_rep_c2446_3082e, which fitted to a divergent selection pattern (Table S2). The former was located within the E3 ubiquitin‐protein ligase gene, while the second was within a gene annotated as collagen alpha‐1(IV) chain. The most consistent outlier was Oed_rep_c2446_3082e, detected in the three ARL‐scenarios at *p* < 0.05, while Oed_rep_c4836_2869e was only detected in the ARL_HIER_ scenario.

### 3.3 Signals of selection for ARRAY markers

The number of outliers detected with BAYESCAN was much lower than those detected with ARLEQUIN in both the individual (BY_IND_ vs. ARL_IND_) and pooled (ARL_POOL/HIER_ and BY_POOL_) scenarios, suggesting a certain proportion of false positives with ARLEQUIN (Table 2). Accordingly, we considered the most consistent outliers with ARLEQUIN those detected at *p* < 0.01. Outliers for balancing selection were exclusively detected with ARLEQUIN, and the high number, between 205 and 1,023 (*p* < 0.01), was in accordance with previous reports suggesting a notable proportion of false positives with this program when checking for balancing selection (Narum & Hess, 2011). Figures for divergent selection, the most important model to be tested in our study regarding bonamiosis resistance, ranged from 24 to 197 with BAYESCAN (BY_POOL_ and BY_IND_, respectively) and from 345 for AR_POOL_ to 1,226 for ARL_IND_ with ARLEQUIN (*p* < 0.01). On the other hand, a higher number of outliers were detected with ARL_HIER_ than with ARL_POOL_, when using pooling strategies, although these figures were very similar for divergent selection (*p* < 0.01:355 vs. 345).

**Table 2:** Detection of outlier loci using two scenarios with BAYESCAN (BY_IND_, including all beds individually; BY_POOL_, pooling the beds by bonamiosis resistance) at FDR < 0.01 and three scenarios with ARLEQUIN (ARL_IND_, including all beds individually; ARL_HIER_, using a hierarchical island model where the beds were grouped by bonamiosis resistance; ARL_POOL_, pooling the beds by bonamiosis resistance) at *p* < 0.01

Model	Outliers		
	Divergent	Balancing	Total
BY_IND_	197	0	197
BY_POOL_	24	0	24
ARL_IND_	596 (1,226)	1,023 (3,088)	1,619 (4,314)
ARL_HIER_	355 (899)	463 (1,627)	818 (2,526)
ARL_POOL_	345 (803)	205 (731)	550 (1,534)

Note

In parentheses, number of suggestive outliers with ARLEQUIN (*p* < 0.05).

**Figure 2:**
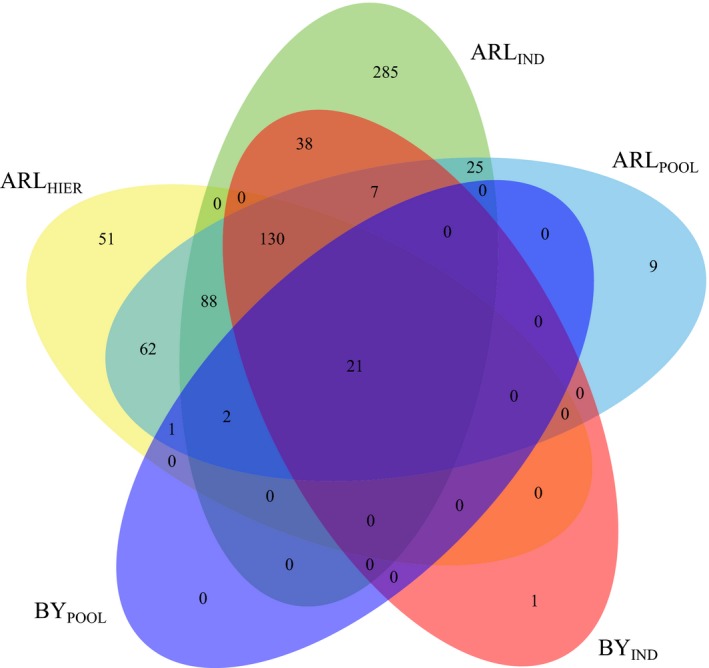
Venn diagram showing the correspondence among the different statistical approaches (ARL, ARLEQUIN; BY, BAYESCAN) and scenarios used for detection of outlier loci (ARL_HIER_, using a hierarchical island model where the beds are grouped by their bonamiosis resistance; BY_IND_ and ARL_IND_, including individually all beds; BY_POOL_ and ARL_POOL_, pooling the beds according to their bonamiosis resistance). Only divergent outliers with BAYESCAN at FDR < 0.05 and ARLEQUIN at *p* < 0.01 were included

The correspondence between the BAYESCAN and ARLEQUIN (*p* < 0.01) strategies outlined previously is shown in the Venn diagram shown in Figure 2. Most outliers detected in pooling scenarios were also detected in the individual ones, especially with BAYESCAN, where 21 out of 24 outliers detected with BY_POOL_ were also detected with BY_IND_ (87.5%). With ARLEQUIN, 273 out of 396 outliers detected with pooling strategies (ARL_POOL_ ARL_HIER_) were also detected with ARL_IND_ (68.9%). This is an expected result since, hypothetically, the individual scenario should detect outliers for most if not all selective factors. A total of 21 markers were consistently detected as divergent outliers in the five scenarios considered, including pooling strategies, and thus, we considered this marker set along with the CAND Oed_rep_c2446_3082e as the 22 most consistent outlier loci putatively associated with bonamiosis resistance in our study (strict (S) criterion, denoted outlier_POOL‐S_). Additionally, we considered other less strict, although consistent panel, including the CAND Oed_rep_c2446_3082e, all loci detected with BY_POOL_ (24 loci) and the intersection of ARLEQUIN pooling strategies at *p* < 0.01 (ARL_POOL_ ∩ ARL_HIER_; 62 loci), which rendered a total of 87 outliers (less strict (L) criterion, denoted outlier_POOL‐L_).

### 3.4 Genomic information of candidate outliers: linkage disequilibrium (LD) and gene mining

Considering the density of the flat oyster SNP panel, we hypothesized that some outlier loci may be located at the same genomic region associated with divergence between LTA and NV populations. This would be especially true if these regions (QTLs) showed a strong effect on bonamiosis resistance, determining a significant selective sweeping around the favorable mutations. Thus, we checked for linkage disequilibrium (LD) among the 22 outlier_POOL‐S_ loci considering all oyster samples as a single population, but also within each of the six populations evaluated. There is a trade‐off when using these strategies; using the whole dataset, LD is increased due to admixture, and thus, we have lower precision for pinpointing associated genomic regions. However, it has the advantage of increasing sample size and, thus, the statistical power to detect associations; however, testing LD in populations at HWE enables fine mapping of the associated genomic regions, but it has the disadvantage of decreasing sample size and therefore statistical power. Due to the lower structure of flat oyster populations and the small sample size (~16) in our study, the first strategy should show higher resolution. Additionally, we performed the same analysis with 50 randomly selected loci from the whole 14,065 loci to be taken as an unlinked dataset reference. Using the 22 outlier_POOL‐S_, a total of 206 significant LD tests were detected out of 231 performed (*p* < 0.05; 89.2%), 173 after Bonferroni correction (*p* < 0.00022; 74.9%), which strongly supports linkage among several of these loci (Table S3). When using the reference of 50 randomly selected unlinked loci in the whole population data, 56 significant LD tests were detected at *p* < 0.05 out of 903 performed (6.2%) and 3 after Bonferroni correction (0.3%). A LD pairwise matrix was constructed for integrating data, and strong linkage signals were observed among 19 loci (161,992, 162,137, 163,443, 163,478, 165,368, 168,827, 168,879, 172,878, 174,144, 174,273, 179,850, 183,021, 183,584, 183,747, 184,858, 186,592, 186,978, 199,647, and 203,903), putatively constituting a single QTL related to flat oyster resistance to bonamiosis (Table 3). Even the three remaining loci (168,138, 174,715, and Oed_rep_c2446_3082e) showed weak signals of linkage with the main outlined cluster, suggesting location in the vicinity. This observation was also confirmed at population level, and although the figures were notably lower than those with the whole dataset (average LD at *p* < 0.05: ~ 40%; average after Bonferroni correction: ~12%) (Table 4) due to the lower statistical power, the proportion of LD was still much higher than the unlinked reference and involved the most consistent set of loci detected with the whole data as a single population (Table S4). We also checked for LD among the 87 outlier_POOL‐L_ loci being the figures lower than with the 22 outlier_POOL‐S_ SNP panel, although again much higher than those expected by chance. A total of 1,861 significant tests were detected over 3,741 (*p* < 0.05; 49.7%) and 633 after Bonferroni correction (16.9%) (Table S5). The most consistent SNPs detected using the whole dataset as a single population were also detected at population level as in the previous case with the 22 outlier_POOL‐S_ SNP panel (Table S6). A set of nine markers among the 65 additional SNPs in this panel (discounting the 22 outlier_POOL‐S_ SNPs) showed strong signals of LD. However, when this information was integrated with the most consistent 19 outlier_POOL‐S_ loci outlined before, they showed strong linkage signals (Table S7), thus supporting the existence of a single major QTL associated with bonamiosis resistance.

**Table 3:** Consistent pairwise linkage disequilibria involving the most strict 22 outlier_POOL‐S_ loci related to bonamiosis resistance using the whole population data. Significant Bonferroni *p* < 0.00013, 0.00013 < *p *< 0.001, 0.001 < *p *< 0.01, and 0.01 < *p *< 0.05 values are shown in red, yellow, green, and blue color, respectively

	162,137	163,443	163,478	165,368	168,138	168,827	168,879	172,878	174,144	174,273	174,715	179,850	183,021	183,584	183,747	184,858	186,592	186,978	199,647	203,903	Oed_rep_
161,992	0	0	0	0	0.0059	0	0	0	0	0	0.0135	0	0	0	0	0	7E‐05	0	0	0	0.01564
162,137		0	0	0	0.0564	0	0	0	0	0	0.0167	0	0	0	0	0	0	0	0	0	0.48507
163,443			0	0	0.0685	0	0	0	0	0	0.07	0	0	0	0	0	0	0	0	0	0.12944
163,478				0	0.1992	0.0007	0	0	0	0	0.0073	0	0	0	0	0	0.0119	0	0.0142	0	0.50748
165,368					0.0751	0	0	0	0	0	5E‐05	0	0	0	0	0	0.002	0	0	0	0.01009
168,138						0.0592	0.0437	0.0694	0.0162	0.0262	0.0251	0.3049	0.0062	0.0442	0.052	0.0094	0.3515	0.04	0.0026	0.1326	0.00117
168,827							0	0	0	0	0.0316	0	0	0	0	0	0.1581	0	0	0	0.1233
168,879								0	0	0	0.0084	0	0	0	0	0	0	0	0	0	0.54063
172,878									0	0	0.031	0	0	0	0	0	0	0	0	0	0.2193
174,144										0	0.0001	0	0	0	0	0	0	0	0	0	0.01137
174,273											0.0017	0	0	0	0	0	0	0	0	0	0.33019
174,715												0.015	0.0193	0.001	0.0234	0.0296	0.0468	0.04	0.0064	0.136	0.02051
179,850													0	0	0	0	0	0	0	0	0.00471
183,021														0	0	0	0	0	0	0	0.00298
183,584															0	0	0.001	0	0	0	0.14131
183,747																0	0	0	0	0	0.06936
184,858																	0.0019	0	1E‐05	0	0.03957
186,592																		0	0.1226	0	0.00212
186,978																			0	0	0.18308
199,647																				0	0.00928
203,903																					0.41023

**Table 4:** Linkage disequilibrium involving the most strict 22 outlier_POOL‐S_ loci related to bonamiosis resistance within populations

Population	No test	Bonferroni	*p* < 0.001	*p* < 0.01	*p* < 0.05	Tot (*p* < 0.05)
Rossmore	229	22	32	27	20	101
	%	9.6	14.0	11.8	8.7	44.1
Ortigueira	210	9	8	21	44	82
	%	4.3	3.8	10.0	21.0	39.0
Quiberon	210	20	11	18	29	78
	%	10.0	4.8	9.5	14.3	37.1
Limfjord	44	15	NA	0	8	23
	%	34.1	NA	0.0	13.6	52.3
Loch Ryan	189	14	30	29	7	80
	%	7.4	15.9	15.3	3.7	42.3
Tralee Bay	190	8	11	13	6	38
		4.2	5.8	6.8	3.2	20.5

DNA sequences comprising these 86 ARRAY outliers were compared against the available *O*. *edulis* and *Crassostrea *spp transcriptomic and genomic databases for their annotation and mining. There were only four markers for which significant alignment with the flanking sequence was identified (Table S8). The locus 165,368, identified in the three genomic databases used, was related to an uncharacterized protein (LOC111134422) of *C. virginica* and located within the scaffold 1,857 of the *C. gigas* genome. The only annotated significant hit corresponded to the locus 173,480, included in the 87 outlier_POOL‐L_ ARRAY set, which matched to a WD repeat (WDR)‐containing protein 5‐like gene. WDRs have been related to many biological functions like signal transduction, transcription regulation, and apoptosis (Li & Roberts, 2001).

### 3.5 Testing outlier loci in different grouping scenarios: bonamiosis resistance vs. other environmental factors

**Figure 3:**
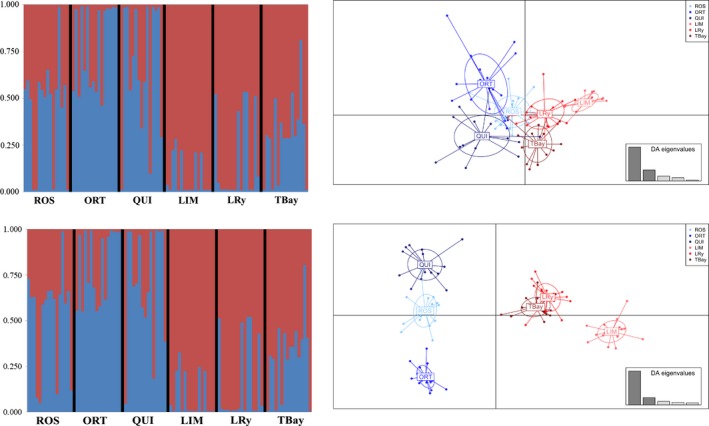
Population structure of flat oyster beds analyzed with STRUCTURE (left part) and Discriminant Analysis of Principal Components (DAPC) (right part) including the 22 most consistent outlier_POOL‐S_ loci regarding bonamiosis resistance (upper part) and the 87 outlier_POOL‐L_ loci following a more relaxed criterion including all BY_POOL_ (24), the Oed_rep_c2446_3082e CAND marker, and those shared by ARL_POOL_ AND ARL_HIER_ at *p* < 0.01 (62) (bottom part). For STRUCTURE analysis, each vertical bar represents one individual and its color proportion the posterior probability for assignment of each individual to the different clusters (*K* = 2) inferred by the program. Population codes are shown in Table 1. For DAPC analysis, weight of retained discriminant analysis (DA) eigenvalues representing >90% of the variance is shown in the bottom right

**Figure 4:**
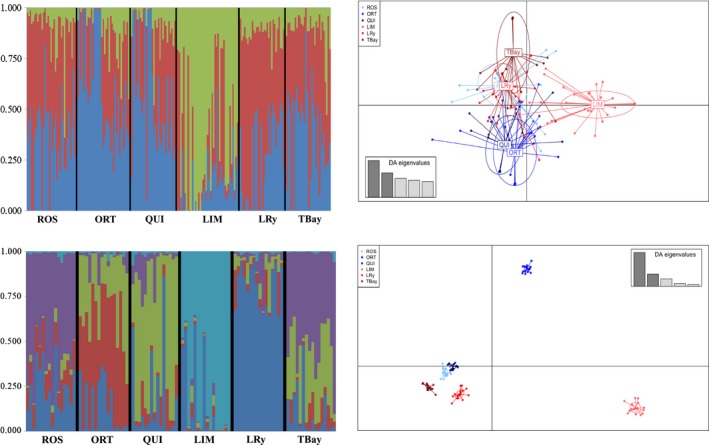
Population structure of flat oyster beds analyzed with STRUCTURE (left part) and Discriminant Analysis of Principal Components (DAPC) (right part) including the whole dataset (upper part) and the most consistent 324 outlier_IND_ loci (BAYESCAN FDR < 0.05; ARLEQUIN *p* < 0.01) not overlapping with pooling strategies (bottom part). For STRUCTURE analysis, each vertical bar represents one individual and its color proportion the posterior probability for assignment of each individual to the different clusters (*K* = 3 and *K* = 5 for the whole dataset and the most consistent 324 outlier_IND_ loci, respectively) inferred by the program. Population codes are shown in Table 1. For DAPC analysis, weight of retained discriminant analysis (DA) eigenvalues representing >90% of the variance is shown

We compared pairwise *F*_ST_ values between the six oyster populations using the panels of 22 and 87 outlier_POOL_ loci (Table 5) with regard to the whole 14,065 SNP dataset, basically representing the neutral background (Table 6). As expected, average figures were much higher with the 22 (average: 0.221; range: 0.027–0.593) and 87 (average: 0.145; range: 0–0.379) SNP panels than with the whole SNP dataset (average: 0.006; range: −0.017 to 0.029). Several pairwise *F*_ST_ values were not significant when using the whole SNP dataset, while nearly all were significant with the 22 and 87 SNP panels, especially between LTA and NV groups. Bayesian clustering with the 22 loci and 87 outlier_POOL‐L_ loci panels rendered in both cases *K* = 2 (Ln P (*K* = 1) = −2419.74; *p *= 0.000) as the most consistent hypothesis, which agrees with the existence of two genomic clusters relating to bonamiosis resistance; one of the clusters was predominant in the LTA group, while the other was in the NV one (Figure 3), and this difference was slightly higher with the 87 outlier_POOL‐L_ SNP panel. This differentiation between both groups with both outlier_POOL_ panels was confirmed with DAPC analyses, but divergence was more evident with the 87 SNP panel, although intra‐group differentiation also increased. Conversely, when the whole SNP dataset was evaluated, the number of population units (*K*) suggested by STRUCTURE following the Evanno's approximation was *K* = 3 (Ln P (*K* = 1) = −1082309.4; *p* = 0.000) and the DAPC showed these three groups spatially segregated (Figure 4).

**Table 5:** Pairwise *F*_ST_ values between flat oyster populations using the most strict set of 22 outlier_POOL‐S_ loci ((BY_POOL_ ∩ ARL_POOL_ ∩ ARL_HIER_) + Oed_rep_c2446_3082e; below diagonal) and those 87 outlier_POOL‐L_ loci including Oed_rep_c2446_3082e, all loci detected with BY_POOL_ (24 loci) and the intersection of ARLEQUIN pooling strategies at *p* < 0.01 (ARL_POOL_ ∩ ARL_HIER_; 62 loci) (above diagonal)

Population	ROS	ORT	QUI	LIM	LRy	TBay
ROS		0.0767**	0.0237	0.2117**	0.1123**	0.0629**
ORT	0.1752**		−0.0000	0.3793**	0.2917**	0.2010**
QUI	0.0614*	0.0272		0.3151**	0.2204**	0.1324**
LIM	0.2599**	0.5925**	0.4677**		0.0348*	0.0754**
LRy	0.0829*	0.4334**	0.3039**	0.0460		0.0385*
TBay	0.0564*	0.3477**	0.2059**	0.1750**	0.0808*	

Note

Population codes come from Table 1.

* *p* < 0.05.

** *p* < 0.001.

**Table 6:** Pairwise *F*_ST_ values between populations using the whole SNP dataset (below diagonal) and the most consistent 324 outlier_IND_ loci (above diagonal)

	ROS	ORT	QUI	LIM	LRy	TBay
ROS		0.1241**	0.1000**	0.3212**	0.0669**	0.0567**
ORT	0.0191**		0.0467**	0.3321**	0.0870**	0.0804**
QUI	0.0004	−0.0029		0.3103**	0.0708**	0.0572**
LIM	0.0281**	0.0287**	−0.0174		0.3188**	0.3449**
LRy	−0.0024	0.0041	0.0067	−0.0054		0.0757**
TBay	0.0007	0.0271**	0.0075*	−0.0087	0.0026	

Note

Population codes are shown in Table 1.

* *p* < 0.05.

** *p* < 0.001.

Finally, we tried to identify a set of outliers hypothetically related to other environmental factors that could enhance genetic divergence between populations studied. The idea was to check for the existence of other factors shaping flat oyster genome divergence in the Atlantic area, but especially to identify a panel of SNPs capable of discriminating oyster populations for practical management. Thus, we selected those outliers exclusively detected in the individual scenarios discounting those detected with BAYESCAN and ARLEQUIN pooling strategies, and a total of 324 SNPs were identified (ARL_IND_: 285, BY_IND_: 1, ARL_IND_ ∩ BY_IND_: 38; outliers_IND_ onwards). The number of population units (*K*) suggested by STRUCTURE in this case was *K* = 5, but each of the six populations showed a unique composition (Figure 4). This was reflected by the DAPC analysis where each population appeared spatially segregated, being ORT and LIM farther apart from all other samples (Figure 4). Furthermore, assignment tests using this panel performed with GENECLASS allocated 100% individuals to their populations (Table S9).

### 3.6 A practical tool for allocating individuals to their populations

**Figure 5:**
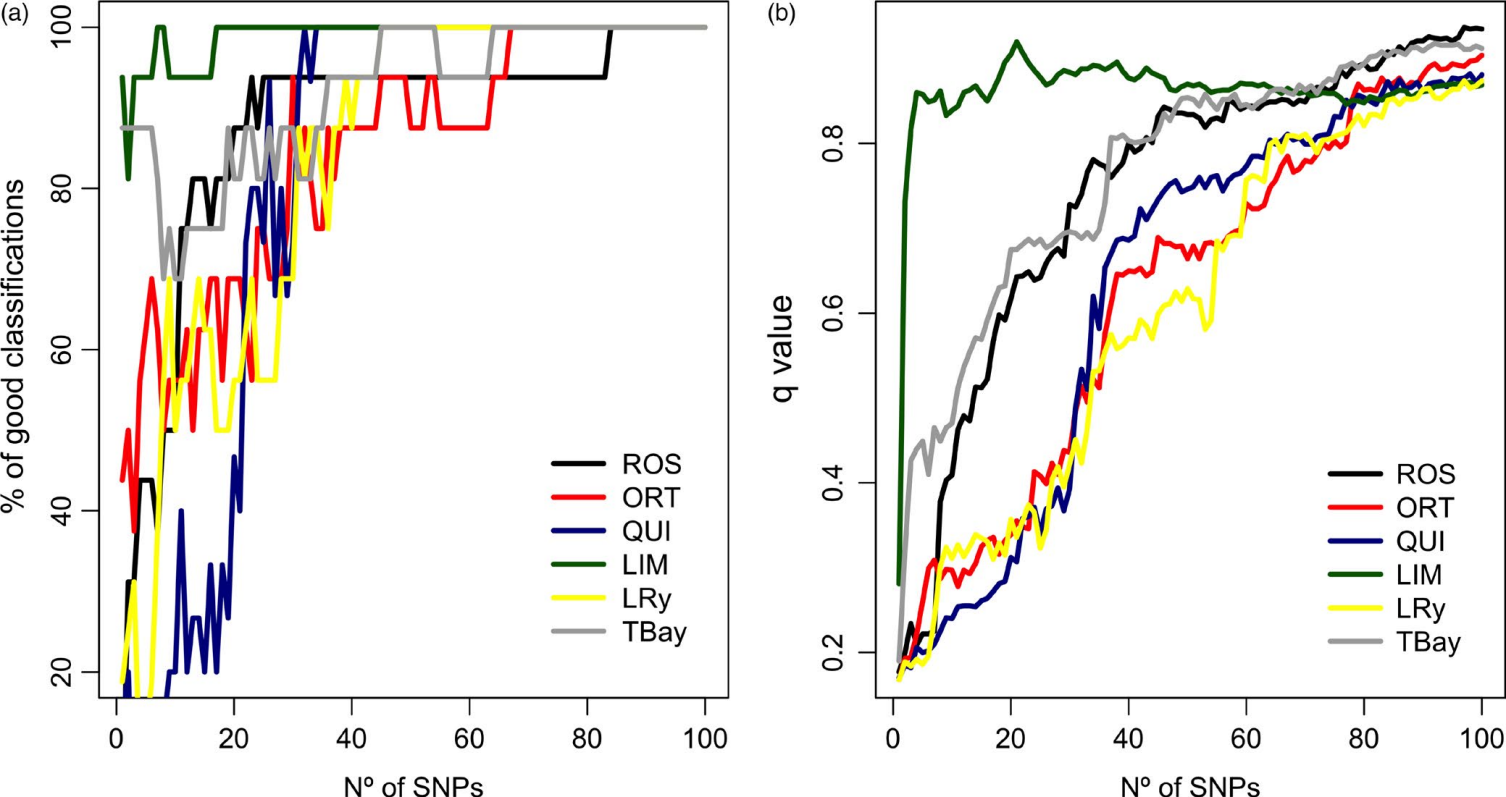
Potential of the 324 outliers_IND_ loci to discriminate flat oyster populations. The outliers_IND_ SNPs were ranked from higher to lower *F*_ST_ and, using a progressive higher number of SNPs of decreasing resolution, checked the proportion of individuals correctly allocated to their population using the admixture coefficient *q*‐value (a) and the mean *q*‐value for each of the populations studied (b)

The sampling design in our study was thought to identify SNP outliers related to bonamiosis resistance following a population genomics approach. Therefore, despite signals for selection related to other unknown environmental factors were detected, the correlation between genetic and environmental data (for instance SEASCAPE analysis) was out of the scope of our work. Furthermore, one of the populations evaluated (ROS) has been subjected to artificial selection for several generations, and thus, it would not be suitable for this analysis. However, the capability of outliers_IND_ to distinguish populations or to assign individuals to populations was outstanding, as outlined before, and we decided to check for the ability of a short SNP panel to correctly allocate individuals to their populations as a practical tool for resources management. Thus, we ranked the 324 outliers_IND_ SNPs from higher to lower *F*_ST_ and, using a progressive higher number of SNPs of decreasing resolution, checked the proportion of individuals correctly allocated to their population using the admixture coefficient *q*‐value (Figure 5a) and the mean *q*‐value for each of the populations studied (Figure 5b). As shown, all individuals were correctly classified using 84 SNPs, and excluding ORT and QUI, 45 SNPs were more than enough to correctly classify the remaining populations. On the other hand, the mean *q*‐value approached asymptotically to the maximum (>0.9), excluding LIM, which reached nearly the maximum *q*‐value with only five SNPs.

## 4 DISCUSSION

European flat oyster production is severely threatened by *B*. *ostreae* in the European Atlantic area, and despite the connectivity among oyster populations mainly through larvae advection (Vera et al., 2016), the presence of this parasite is apparently restricted to specific Atlantic areas (Engelsma et al., 2014). We took advantage of the restricted parasite distribution and the low genetic structure of flat oyster in the Atlantic area and selected three LTA and three NV beds from the three different genetic units previously reported (Vera et al., 2016) trying to identify genomic regions showing signatures of selection related to bonamiosis resistance. Genomic signatures for divergent or balancing selection have been reported despite high gene flow between populations (Prado, Vera, Hermida, Bouza, et al., 2018; Vandamme et al., 2014; Vilas et al., 2015). We assumed as a working hypothesis that outlier loci for divergent selection between both oyster groups could underlie QTLs for resistance to the parasite and that selective sweeps around these regions would determine significant differentiation over the neutral background for a set of markers considering the marker density of the screening performed. Similar approaches have been carried out by comparing domestic vs. wild strains or wild populations to identify candidate genomic regions related to selection in crops (Fawcett et al., 2013; Price et al., 2018) or animals (Bradic, Teotonio, & Borowsky, 2013; Hohenlohe et al., 2010; Prado, Vera, Hermida, Blanco, et al., 2018).

A total of 37 validated CAND SNPs and 13,057 polymorphic ARRAY SNPs passed our filtering pipelines and were used to analyze flat oyster populations. Gutierrez et al. (2017) using a very strict filtering criteria discarded a total of 3,798 SNPs in the oyster Axiom platform, but a certain amount of these were retained in our study because they were polymorphic and population parameters (HWE) suggested confidence. Furthermore, the most relevant 87 SNPs related to signatures of selection to bonamiosis passed the filtering criteria in both studies and these SNPs are thus consistently associated with bonamiosis. A total of 310 polymorphic SNPs identified by Gutierrez et al. (2017) were not polymorphic in our study, but in this case, the difference can be mostly attributed to the different sampling scenario, particularly, the sample from Mediterranean Sea analyzed by those authors. The amount of polymorphic markers used in our study represents a density of ~1 SNP/83 kb considering the *C*‐value reported for flat oyster (average 2*C* value = 2.33; Rodríguez‐Juíz, Torrado, & Mendez, 1996).

The global genetic differentiation of flat oyster in the Atlantic area using the 13,057 polymorphic SNPs (*F*_ST_ = 0.0061) was similar to that previously documented using 16 microsatellite loci on a broader sample collection in the same sampling area (*F*_ST_ = 0.0079; Vera et al., 2016), both studies suggesting high connectivity among populations in the Atlantic area. Furthermore, the three population units previously detected using a panel of 16 microsatellite loci (Vera et al., 2016) in the Atlantic region (Spanish, Irish/British/French and Dutch/Danish clusters), which were associated with the existence of oceanic fronts in that region (Blanco et al., 2016), were also detected in the present study with the whole SNP dataset. The only difference detected was the clustering of QUI with ORT (present study) instead of with the British Isles cluster (Vera et al., 2016), but it should be noted that both populations are in the Biscay Bay and pertain to the same OSPAR region (Figure 1); thus, increasing the power of markers might render a different result for QUI, a population in the edge between both regions. This structure has also been described for other mollusk species like the edible cockle (*Cerastoderma edule*) (Martínez, Freire, Arias‐Pérez, Méndez, & Insua, 2015), which confirms general mechanisms of dispersion related to pelagic larvae advection associated with the pattern of Atlantic Ocean currents.

A main issue for recovery of *O. edulis* production and enhancing natural oyster beds would be the identification of genetic markers associated with resistance to *B. ostreae*. Previous studies documented that populations long‐term exposed to the parasite are more resistant than naïve ones (Elston et al., 1987; da Silva et al., 2005) and that breeding programs respond to selection for bonamiosis resistance, which demonstrates the existence of underlying genetic variation for resistance to *B. ostreae* (Lynch et al., 2014; Naciri‐Graven, Martin, Baud, Renault, & Gerard, 1998). Furthermore, some QTLs were detected in preliminary studies at family level (Harrang et al., 2015; Lallias et al., 2009). We applied two complementary strategies to look for genetic markers associated with resistance in a population scenario. On one hand, we focused on makers linked to candidate immune genes (CAND) showing differential expression between LTA and NV oysters previously reported by Ronza et al. (2018), for which 37 out of 77 markers were validated using Sequenom technology. This is a rather low validation success regarding previous reports using a similar approach with anonymous markers (Cruz et al., 2017; Siccha‐Ramírez et al., 2018) and could be related to the use of mature mRNA sequences as a reference for primer design or the highly polymorphic nature of bivalve species causing interference in probe sequence binding (Pino‐Querido et al., 2015). The lack of the whole assembly of the flat oyster genome, which represents an important limitation as discussed below, precluded the location of introns, and thus the adequate positioning of primers for some loci. Among the 37 validated loci, we detected two CAND SNPs evidencing signals for divergent selection between LTA and NV oyster groups, one associated to the E3 ubiquitin‐protein ligase gene and the other to the collagen alpha‐1(IV) chain gene. Sixteen genes related to the ubiquitin–proteasome pathway, including the E3 ubiquitin‐protein ligase here identified, were reported to be differentially expressed (DE) when comparing LTA and NV gene expression profiles, and this pathway showed a significant enrichment regarding the whole immune‐enriched flat oyster transcriptome (Pardo et al., 2016; Ronza et al., 2018). The other gene, collagen alpha‐1(IV) chain, showed signals of divergence with all the ARLEQUIN strategies applied (*p* < 0.05), although not with BAYESCAN. In the study by Ronza et al. (2018), several genes encoding structural extracellular matrix (ECM) proteins, including various collagen isoforms, were DE between LTA and NV oyster groups, and ECM‐related GO terms appeared significantly enriched. These authors emphasized the critical role of ECM as regulator of innate immunity, and especially, as the first external barrier for adhesion and passing through the cell membrane by an intracellular parasite such as *B*. *ostreae*. These genes and markers linked to them should be considered for further studies geared toward increasing resistance to bonamiosis in flat oyster stocks. As a complementary approach, we harnessed the recently developed ~15,000 SNP chip for flat oyster (Gutierrez et al., 2017) to perform a high‐resolution genomic screening with primarily anonymous markers. We identified a set of 21 highly confident SNP markers differentiating flat oyster populations with all strategies applied and 65 additional ones including other loci at *p* < 0.01 mostly with ARL pooling strategies.

By combining CAND and ARRAY SNP information, we defined a set of 22 very consistent outlier loci (1 CAND and 21 ARRAY markers) related to divergent selection between LTA and NV groups taking as reference the neutral background for Atlantic oyster outlined before. Remarkably, most of these markers showed strong LD among them, apparently targeting to a specific genomic region in the flat oyster genome, suggestive of a large QTL as the primary determinant of resistance to bonamiosis. When a less strict, although consistent 87 SNP set was used by adding another 65 SNPs, another cluster of nine loci demonstrated to be linked to that outlined before, providing additional support for the existence of a single major QTL. We looked for annotation of the outlier ARRAY loci using the flat oyster database and the *C*. *gigas* genome, and significant matches and annotation of candidate markers associated to bonamiosis resistance were poor, particularly in the most interesting genomic region identified. Clearly, the lack of the whole genome flat oyster assembly and the low quality of the *C. gigas* genome limited successful annotation, gene mining, and functional enrichment, which strongly recommending the urgency of a draft reference genome for *O*. *edulis.* This genome would greatly facilitate the identification of responsible genes and mutations for achieving a more resistant strain to be used for recovering production and appropriate management of flat oyster resources in the Atlantic area.

Finally, although not the main goal of the study, we could identify a broad set of 324 ourliers_IND_ loci putatively not related to bonamiosis resistance but to abiotic environmental variables. This SNP panel enabled the full differentiation of the six populations studied in the Atlantic area and the correct allocation of all individuals to their populations. Even the very close geographic samples in the British Isles were allocated with 100% accuracy to their original populations. The use of outlier loci for distinguishing populations and successful allocation of individuals to populations has been applied in other studies with aquatic organisms showing low population structuring (Candy et al., 2015; Carreras et al., 2017). Moreover, we estimated the minimum panel of SNPs required for individual allocation using the gene frequencies of the six reference populations and the *q* admixture estimator. A total of 84 SNPs were enough to fully discriminate populations include those very close in the British Isles, but a set of 45 would be more than enough for all the remaining populations more distantly related in the Atlantic area. A very similar result was obtained in the same geographic area in the turbot (*Scophthalmus maximus*), a fish species whose dispersal mechanism is mainly related to larval advection (Prado, Vera, Hermida, Bouza, et al., 2018).

In this study, we used a population genomics approach to identify putative selective sweeps harnessing QTLs related to bonamiosis resistance in flat oyster, the main threat for its production and viability. The combination of candidate gene and genomic screening strategies enabled preliminary ascertainment of the genomic architecture of resistance to bonamiosis, where a putative major QTL appears to explain most of the divergence between LTA and NV populations. Other minor QTLs scattered across the genome, including two candidate genes associated with enriched pathways and functions activated against the parasite, would also explain the divergence between both population sets. The lack of a flat oyster reference genome precluded a high‐resolution analysis genes and genetic variants associated with resistance, which remains an urgent task for the near future. In any case, the information obtained provides a set of markers to be evaluated as tools to enhance selective breeding programs and for appropriate management and recovery of flat oyster beds.

## CONFLICT OF INTEREST

None declared.

## Data Availability

Data for this study are available at Dryad Digital Repository: https://doi.org/10.5061/dryad.89bv2s6
